# 2-Ammonio-3-(4-nitro­phen­yl)propanoate monohydrate

**DOI:** 10.1107/S1600536808017960

**Published:** 2008-07-09

**Authors:** Wei Dai, Da-Wei Fu

**Affiliations:** a Ordered Matter Science Research Center, College of Chemistry and Chemical Engineering, Southeast University, Nanjing 210096, People’s Republic of China

## Abstract

The title compound, C_9_H_10_N_2_O_4_·H_2_O, exists as a zwitterion with a deprotonated carboxyl group and a protonated amine group. The crystal packing is stabilized by inter­molecular N—H⋯O and O—H⋯O hydrogen bonds, building sheets parallel to the (001) plane.

## Related literature

For details on α-amino acids as precursors for the synthesis of novel biologically active compounds, see: Lucchese *et al.* (2007 ▶); Arki *et al.* (2004 ▶); Hauck *et al.* (2006 ▶); Azim *et al.* (2006 ▶).
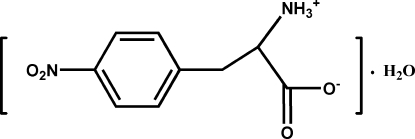


## Experimental

### 

#### Crystal data

C_9_H_10_N_2_O_4_·H_2_O
*M* *_r_* = 228.21
Monoclinic, 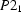

*a* = 6.2349 (12) Å
*b* = 5.2990 (11) Å
*c* = 15.727 (3) Å
β = 101.40 (3)°
*V* = 509.35 (18) Å^3^
*Z* = 2
Mo *K*α radiation
μ = 0.12 mm^−1^
*T* = 293 (2) K
0.30 × 0.25 × 0.15 mm

#### Data collection

Rigaku Mercury2 diffractometer
Absorption correction: multi-scan (*CrystalClear*; Rigaku, 2005 ▶) *T*_min_ = 0.964, *T*_max_ = 0.982
5388 measured reflections
1297 independent reflections
1184 reflections with *I* > 2σ(*I*)
*R*_int_ = 0.034

#### Refinement

*R*[*F* ^2^ > 2σ(*F* ^2^)] = 0.051
*wR*(*F* ^2^) = 0.140
*S* = 1.14
1297 reflections
153 parameters
1 restraint
H atoms treated by a mixture of independent and constrained refinement
Δρ_max_ = 0.36 e Å^−3^
Δρ_min_ = −0.34 e Å^−3^

### 

Data collection: *CrystalClear* (Rigaku, 2005 ▶); cell refinement: *CrystalClear*; data reduction: *CrystalClear*; program(s) used to solve structure: *SHELXS97* (Sheldrick, 2008 ▶); program(s) used to refine structure: *SHELXL97* (Sheldrick, 2008 ▶); molecular graphics: *SHELXTL* (Sheldrick, 2008 ▶); software used to prepare material for publication: *SHELXL97*.

## Supplementary Material

Crystal structure: contains datablocks I, global. DOI: 10.1107/S1600536808017960/rz2221sup1.cif

Structure factors: contains datablocks I. DOI: 10.1107/S1600536808017960/rz2221Isup2.hkl

Additional supplementary materials:  crystallographic information; 3D view; checkCIF report

## Figures and Tables

**Table 1:** Hydrogen-bond geometry (Å, °)

*D*—H⋯*A*	*D*—H	H⋯*A*	*D*⋯*A*	*D*—H⋯*A*
N2—H2*A*⋯O3^i^	0.89	2.16	2.745 (4)	123
N2—H2*B*⋯O4^ii^	0.89	2.30	2.904 (4)	125
O5—H30⋯O4^iii^	0.92 (6)	1.81 (6)	2.721 (5)	177 (5)
O5—H31⋯O3^iv^	0.79 (8)	2.11 (8)	2.809 (4)	148 (6)

Symmetry codes: (i) 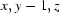; (ii) 
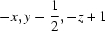
; (iii) 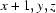; (iv) 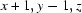.

**Fig. 1.**
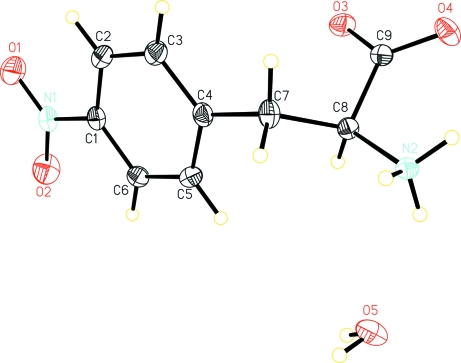
A view of the title compound with the atomic numbering scheme. Displacement ellipsoids were drawn at the 30% probability level.

**Fig. 2.**
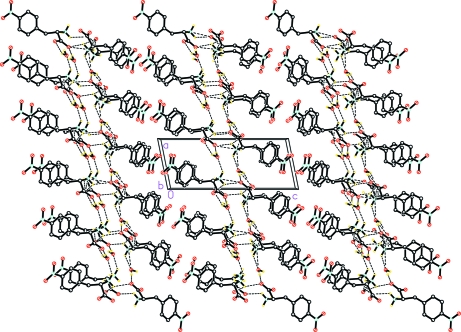
The crystal packing of the title compound viewed along the b axis. All hydrogen atoms not involved in hydrogen bonding (dashed lines) are omitted for clarity.

## supplementary crystallographic information

### Comment

α-Amino acids are important molecules due to their pharmacological properties. Recently, there has been an increased interest in the enantiomeric preparation of α-amino acids as precursors for the synthesis of novel biologically active compounds (Lucchese *et al.*, 2007; Arki *et al.*, 2004; Hauck *et al.*, 2006; Azim *et al.*, 2006). Here we report the synthesis and crystal structure of the title compound.

The title compound exists as a zwitter ion with a deprotonated carboxyl group and a protonated amino group (Fig. 1). It crystallizes with one water molecule in the asymmetric unit. The crystal packing is stabilized by intermolecular N—H···O and O—H···O hydrogen bonds building sheets parallel to the (001) plane (Table 1, Fig. 2).

### Experimental

Under nitrogen protection, 2-amino-3-phenylpropanoic acid (30 mmol), nitric acid (50 mmol) and sulfuric acid (20 mmol) were added in a flask. The mixture was stirred at 110 °C for 3 h. The resulting solution was poured into ice water (100 ml), then filtered and washed with distilled water. The crude product was recrystallized with distilled water to yield colorless block-like crystals, suitable for X-ray analysis.

### Refinement

All H atoms attached to C atoms and N atom were placed geometrically and treated as riding with C—H = 0.98 Å (methine), 0.97 Å (methylene), 0.93 Å (aromatic) and N—H = 0.89 Å and with *U*_iso_(H) = 1.2*U*_eq_(C) or 1.5*U*_eq_(N). H atoms of water molecule were located in difference Fourier maps and refined freely. In the absence of significant anomalous scattering, the absolute configuration could not be reliably determined and then the Friedel pairs were merged.

### Crystal data

**Table:**

C_9_H_10_N_2_O_4_·H_2_O	*F*_000_ = 240
*M**_r_* = 228.21	*D*_x_ = 1.488 Mg m^−^^3^
Monoclinic, *P*2_1_	Mo *K*α radiation λ = 0.71073 Å
Hall symbol: P 2yb	Cell parameters from 1458 reflections
*a* = 6.2349 (12) Å	θ = 3.3–27.5º
*b* = 5.2990 (11) Å	µ = 0.12 mm^−^^1^
*c* = 15.727 (3) Å	*T* = 293 (2) K
β = 101.40 (3)º	Block, colourless
*V* = 509.35 (18) Å^3^	0.30 × 0.25 × 0.15 mm
*Z* = 2	

### Data collection

**Table:**

Rigaku Mercury2 (2x2 bin mode) diffractometer	1297 independent reflections
Radiation source: fine-focus sealed tube	1184 reflections with *I* > 2σ(*I*)
Monochromator: graphite	*R*_int_ = 0.034
Detector resolution: 13.6612 pixels mm^-1^	θ_max_ = 27.5º
*T* = 293(2) K	θ_min_ = 3.3º
ω scans	*h* = −8→8
Absorption correction: multi-scan(CrystalClear; Rigaku, 2005)	*k* = −6→6
*T*_min_ = 0.964, *T*_max_ = 0.982	*l* = −20→19
5388 measured reflections	

### Refinement

**Table:**

Refinement on *F*^2^	Secondary atom site location: difference Fourier map
Least-squares matrix: full	Hydrogen site location: inferred from neighbouring sites
*R*[*F*^2^ > 2σ(*F*^2^)] = 0.051	H atoms treated by a mixture of independent and constrained refinement
*wR*(*F*^2^) = 0.140	*w* = 1/[σ^2^(*F*_o_^2^) + (0.0664*P*)^2^ + 0.2902*P*] where *P* = (*F*_o_^2^ + 2*F*_c_^2^)/3
*S* = 1.14	(Δ/σ)_max_ < 0.001
1297 reflections	Δρ_max_ = 0.36 e Å^−^^3^
153 parameters	Δρ_min_ = −0.34 e Å^−^^3^
1 restraint	Extinction correction: none
Primary atom site location: structure-invariant direct methods	

### Special details

**Table:**

Geometry. All e.s.d.'s (except the e.s.d. in the dihedral angle between two l.s. planes) are estimated using the full covariance matrix. The cell e.s.d.'s are taken into account individually in the estimation of e.s.d.'s in distances, angles and torsion angles; correlations between e.s.d.'s in cell parameters are only used when they are defined by crystal symmetry. An approximate (isotropic) treatment of cell e.s.d.'s is used for estimating e.s.d.'s involving l.s. planes.
Refinement. Refinement of *F*^2^ against ALL reflections. The weighted *R*-factor *wR* and goodness of fit *S* are based on *F*^2^, conventional *R*-factors *R* are based on *F*, with *F* set to zero for negative *F*^2^. The threshold expression of *F*^2^ > σ(*F*^2^) is used only for calculating *R*-factors(gt) *etc*. and is not relevant to the choice of reflections for refinement. *R*-factors based on *F*^2^ are statistically about twice as large as those based on *F*, and *R*- factors based on ALL data will be even larger.

### Fractional atomic coordinates and isotropic or equivalent isotropic displacement parameters (Å^2^)

**Table:**

	*x*	*y*	*z*	*U*_iso_*/*U*_eq_	
O1	0.3398 (5)	0.1706 (7)	0.0174 (2)	0.0512 (9)	
N2	0.2068 (5)	−0.9188 (6)	0.41031 (19)	0.0286 (7)	
H2A	0.2056	−1.0468	0.3736	0.043*	
H2B	0.0991	−0.9385	0.4394	0.043*	
H2D	0.3345	−0.9154	0.4475	0.043*	
O3	−0.0544 (4)	−0.3216 (6)	0.34808 (17)	0.0342 (6)	
C1	0.3833 (6)	−0.1391 (8)	0.1227 (2)	0.0283 (8)	
C7	0.1506 (6)	−0.7322 (7)	0.2635 (2)	0.0301 (8)	
H7A	0.2309	−0.8847	0.2560	0.036*	
H7B	−0.0026	−0.7631	0.2392	0.036*	
O4	−0.1341 (4)	−0.6381 (6)	0.42852 (18)	0.0367 (7)	
O2	0.6547 (5)	0.1366 (7)	0.1010 (2)	0.0537 (9)	
C5	0.4472 (6)	−0.4434 (9)	0.2352 (2)	0.0346 (9)	
H5A	0.5413	−0.5221	0.2807	0.042*	
C9	−0.0204 (5)	−0.5355 (7)	0.3814 (2)	0.0243 (7)	
C2	0.1690 (6)	−0.2143 (9)	0.0984 (2)	0.0335 (9)	
H2C	0.0766	−0.1372	0.0520	0.040*	
C3	0.0930 (6)	−0.4068 (9)	0.1441 (2)	0.0330 (8)	
H3A	−0.0516	−0.4600	0.1282	0.040*	
N1	0.4628 (5)	0.0705 (7)	0.0771 (2)	0.0351 (8)	
C8	0.1756 (5)	−0.6791 (7)	0.3613 (2)	0.0231 (7)	
H8A	0.3065	−0.5751	0.3798	0.028*	
C6	0.5246 (6)	−0.2510 (9)	0.1903 (2)	0.0334 (9)	
H6A	0.6694	−0.1982	0.2055	0.040*	
C4	0.2310 (6)	−0.5208 (8)	0.2133 (2)	0.0281 (7)	
O5	0.6393 (5)	−1.0768 (7)	0.4286 (2)	0.0412 (7)	
H30	0.715 (8)	−0.930 (12)	0.427 (3)	0.047 (14)*	
H31	0.681 (11)	−1.158 (18)	0.393 (4)	0.08 (2)*	

### Atomic displacement parameters (Å^2^)

**Table:**

	*U*^11^	*U*^22^	*U*^33^	*U*^12^	*U*^13^	*U*^23^
O1	0.0582 (19)	0.050 (2)	0.0455 (16)	0.0021 (17)	0.0115 (14)	0.0193 (17)
N2	0.0280 (14)	0.0290 (16)	0.0305 (14)	0.0084 (13)	0.0095 (11)	0.0063 (14)
O3	0.0334 (13)	0.0275 (14)	0.0409 (14)	0.0068 (12)	0.0057 (11)	0.0025 (13)
C1	0.0351 (17)	0.0278 (19)	0.0241 (15)	−0.0002 (15)	0.0111 (13)	−0.0019 (15)
C7	0.0401 (19)	0.0273 (19)	0.0246 (16)	−0.0030 (16)	0.0109 (14)	−0.0022 (15)
O4	0.0374 (14)	0.0334 (15)	0.0452 (14)	0.0024 (13)	0.0223 (12)	−0.0027 (13)
O2	0.0545 (19)	0.051 (2)	0.0563 (18)	−0.0224 (17)	0.0128 (15)	0.0021 (17)
C5	0.0274 (17)	0.044 (2)	0.0313 (17)	0.0054 (18)	0.0037 (14)	0.0094 (18)
C9	0.0244 (15)	0.0246 (17)	0.0231 (14)	0.0003 (14)	0.0031 (12)	−0.0061 (14)
C2	0.0333 (18)	0.041 (2)	0.0249 (16)	0.0008 (17)	0.0024 (14)	0.0034 (17)
C3	0.0320 (17)	0.040 (2)	0.0263 (16)	−0.0036 (18)	0.0037 (14)	−0.0015 (17)
N1	0.0443 (18)	0.0350 (18)	0.0289 (15)	−0.0024 (17)	0.0143 (14)	−0.0004 (14)
C8	0.0263 (15)	0.0180 (16)	0.0255 (15)	0.0014 (13)	0.0064 (12)	0.0012 (13)
C6	0.0229 (16)	0.045 (2)	0.0330 (17)	−0.0026 (16)	0.0071 (13)	0.0041 (18)
C4	0.0327 (17)	0.0298 (18)	0.0238 (15)	0.0028 (16)	0.0103 (13)	−0.0024 (16)
O5	0.0308 (14)	0.0402 (19)	0.0557 (18)	0.0035 (14)	0.0160 (13)	−0.0028 (15)

### Geometric parameters (Å, °)

**Table:**

O1—N1	1.211 (4)	O2—N1	1.232 (4)
N2—C8	1.478 (5)	C5—C4	1.385 (5)
N2—H2A	0.8900	C5—C6	1.381 (6)
N2—H2B	0.8900	C5—H5A	0.9300
N2—H2D	0.8900	C9—C8	1.524 (5)
O3—C9	1.249 (5)	C2—C3	1.384 (6)
C1—C6	1.374 (5)	C2—H2C	0.9300
C1—C2	1.374 (5)	C3—C4	1.387 (5)
C1—N1	1.461 (5)	C3—H3A	0.9300
C7—C4	1.512 (5)	C8—H8A	0.9800
C7—C8	1.541 (4)	C6—H6A	0.9300
C7—H7A	0.9700	O5—H30	0.92 (6)
C7—H7B	0.9700	O5—H31	0.79 (8)
O4—C9	1.247 (4)		
			
C8—N2—H2A	109.5	C1—C2—H2C	120.5
C8—N2—H2B	109.5	C3—C2—H2C	120.5
H2A—N2—H2B	109.5	C2—C3—C4	120.4 (3)
C8—N2—H2D	109.5	C2—C3—H3A	119.8
H2A—N2—H2D	109.5	C4—C3—H3A	119.8
H2B—N2—H2D	109.5	O1—N1—O2	122.7 (4)
C6—C1—C2	122.0 (4)	O1—N1—C1	119.3 (3)
C6—C1—N1	118.7 (3)	O2—N1—C1	118.0 (3)
C2—C1—N1	119.3 (3)	N2—C8—C9	110.4 (3)
C4—C7—C8	114.0 (3)	N2—C8—C7	109.9 (3)
C4—C7—H7A	108.7	C9—C8—C7	111.7 (3)
C8—C7—H7A	108.7	N2—C8—H8A	108.2
C4—C7—H7B	108.7	C9—C8—H8A	108.2
C8—C7—H7B	108.7	C7—C8—H8A	108.2
H7A—C7—H7B	107.6	C1—C6—C5	118.6 (3)
C4—C5—C6	120.9 (3)	C1—C6—H6A	120.7
C4—C5—H5A	119.6	C5—C6—H6A	120.7
C6—C5—H5A	119.6	C5—C4—C3	119.2 (4)
O4—C9—O3	125.1 (3)	C5—C4—C7	119.8 (3)
O4—C9—C8	118.6 (3)	C3—C4—C7	121.0 (3)
O3—C9—C8	116.3 (3)	H30—O5—H31	101 (6)
C1—C2—C3	118.9 (3)		
			
C6—C1—C2—C3	0.7 (6)	C4—C7—C8—N2	−150.5 (3)
N1—C1—C2—C3	−177.6 (3)	C4—C7—C8—C9	86.6 (4)
C1—C2—C3—C4	0.2 (6)	C2—C1—C6—C5	−0.5 (6)
C6—C1—N1—O1	180.0 (4)	N1—C1—C6—C5	177.8 (4)
C2—C1—N1—O1	−1.7 (5)	C4—C5—C6—C1	−0.7 (6)
C6—C1—N1—O2	0.6 (5)	C6—C5—C4—C3	1.6 (6)
C2—C1—N1—O2	178.9 (4)	C6—C5—C4—C7	179.9 (4)
O4—C9—C8—N2	−4.9 (4)	C2—C3—C4—C5	−1.3 (6)
O3—C9—C8—N2	175.4 (3)	C2—C3—C4—C7	−179.6 (3)
O4—C9—C8—C7	117.7 (4)	C8—C7—C4—C5	59.3 (5)
O3—C9—C8—C7	−61.9 (4)	C8—C7—C4—C3	−122.4 (4)

### Hydrogen-bond geometry (Å, °)

**Table:**

*D*—H···*A*	*D*—H	H···*A*	*D*···*A*	*D*—H···*A*
N2—H2A···O3^i^	0.89	2.16	2.745 (4)	123
N2—H2B···O4^ii^	0.89	2.30	2.904 (4)	125
O5—H30···O4^iii^	0.92 (6)	1.81 (6)	2.721 (5)	177 (5)
O5—H31···O3^iv^	0.79 (8)	2.11 (8)	2.809 (4)	148 (6)

Symmetry codes: (i) *x*, *y*−1, *z*; (ii) −*x*, *y*−1/2, −*z*+1; (iii) *x*+1, *y*, *z*; (iv) *x*+1, *y*−1, *z*.
